# Toxicological Characterization of GHB as a Performance-Enhancing Drug

**DOI:** 10.3389/fpsyt.2022.846983

**Published:** 2022-04-18

**Authors:** Arianna Giorgetti, Francesco Paolo Busardò, Raffaele Giorgetti

**Affiliations:** ^1^Unit of Legal Medicine, Department of Medical and Surgical Sciences, University of Bologna, Bologna, Italy; ^2^Department of Excellence of Biomedical Sciences and Public Health, “Politecnica delle Marche” University of Ancona, Ancona, Italy

**Keywords:** gamma hydroxybutyrate (GHB), sodium oxybate, performance-enhancing drugs (PEDs), psychoactive performance, forensic toxicology

## Abstract

Performance-enhancing drugs (PEDs) are represented by several compounds used to ameliorate the image, the appearance, or an athletic or non-athletic performance. Gamma-hydroxybutyrate (GHB) is an endogenous molecule first used as anesthetic and then marketed as a nutritional supplement with a wide diffusion in the bodybuilding community. The aim of the present work is to provide a toxicological characterization of the use of GHB as a PED, including the scientific basis for its use, the patterns of use/abuse, and the health risks arising from its consumption in this peculiar recreative setting. A literature search was performed on multiple databases including experimental studies on humans and animals as well as epidemiological reports and forensic case reports/series. Experimental studies demonstrated that the use of GHB as a PED is motivated by the release of growth hormone and the induction of sleep. However, the panel of desired performance-related effects was much wider in real cases and epidemiological studies. Even though the use of GHB among bodybuilders has decreased, its use to enhance some kind of performance, particularly sexual ones or social-communicative ones, as well as means to increase mood and perceived energy, is still common.

## Introduction

The term “image-, appearance- or performance-enhancing drug” (IPED, APED, or PED) is used to indicate a wide range of substances used to improve the appearance, to increase musculature as well as self-confidence and self-esteem, and to enhance or increase an athletic performance, which could be professional or non-professional (1, 2). The World Anti-Doping Agency (WADA) has so far included over 192 drugs and methods among the list of banned PEDs, which include androgenic anabolic steroids (AAS), human growth hormone (GH), insulin-like growth factor-1 (IGF-1), and other hormones, but also recreational drugs, such as stimulants, narcotics, cannabinoids, and prescription medications, such as beta agonists and diuretics (3).

However, the universe of APEDs is much wider and complex. Appearance, weight, and eating concerns, rigid or compulsive practices and exercises, body image disturbances and tendencies toward aesthetic and body enhancement are extremely common in western industrial countries (2, 4). Indeed, the use of legal nutritional supplements has been reported to have a prevalence of 49% in the U.S. population and might precede, especially in adolescents, the use of illicit PEDs (2). Moreover, a polypharmacy consumption pattern, with multiple and different substances used to enhance desired effects or counteract the adverse ones, has been demonstrated (5).

Gamma or γ-hydroxybutyrate (GHB) (C_4_H_8_O_3_) is an endogenous short-chain hydroxylated carboxylic acid, exerting a central nervous system (CNS) depressant action (6). This type of effect is thought to be a consequence of its structural similarity to the neurotransmitter gamma-aminobutyric acid (GABA), of which GHB is both a precursor and a metabolite (6, 7). Besides its Food and Drug Administration (FDA) and European agencies approval as treatment of narcolepsy-associated cataplexy and, in some countries, of alcohol dependence (6), GHB has been marketed from 1980 as a performance-enhancer dietary supplement and sold as a pro-drug in products for the gym. Indeed, performance-enhancing and therapeutic effects of GHB, including sleep, vigilance, and mood enhancement, widely overlap. It has been commonly used by body builders (8–11) who acted as early adopters, enhancement drug use innovators, and anticipated an increase in lean muscle mass due to the GHB-induced release of GH (12–15). This lasted until the year 2000, when GHB was listed in the schedule I drugs with the Rape Drug Prohibition Act (6). However, several research projects worldwide are still evaluating therapeutic and possibly performance-enhancing effects.

Renewed attention to the drug has been recently given to GHB as a club drug in various recreational settings (15, 16), in association with the “chemsex” scene (17, 18) or used for drug-facilitated sexual assaults (19).

The aim of the present work is to provide a literature overview of the scientific evidence underlying the use of GHB as a means to enhance the performances, whether connected to appearance, psychomotor, or athletic ones, and to assess the toxicological characteristics and the health risks arising from a specific use of GHB within the framework of PEDs.

## Materials and Methods

A comprehensive literature search was performed on multiple databases (PubMed, Google Scholar, Scopus, ISI Web of Science) focusing on the effects and the use of GHB as a PED. A free-text search with the keywords was done, by combining “GHB,” or “Gamma hydroxybutyrate,” or “sodium oxybate,” and one of the following terms: “performance/-enhancer/PED,” “GH,” or “growth hormone,” “cortisol,” “body building/enhancement,” “weight loss,” “decreased/reduced appetite,” “better sleep,” “self-confidence,” and “muscle growth/strength/tone.”

Keywords were *a priori* established, but also updated on the basis of the retrieved literature.

Experimental studies, in which previously set and declared GHB doses were administered (20), were included in the present research, prioritizing data from systematic reviews and randomized controlled trials, when available. Both human and animal-based investigations were considered. Data on narcoleptic patients treated with GHB were included, but not randomized controlled trials demonstrating the therapeutical effects of sodium oxybate in these patients. Sexual performances were not specifically targeted by our search and only included when collaterally found.

Studies focused on psychomotor performances already included in the previous paper (20) were not considered, as well as articles specifically focusing on driving abilities.

A second set of information was obtained by epidemiological and forensic case reports/case series of GHB intake.

The search was not restricted to English language documents only, but only papers in which a full-text could be retrieved were included.

Finally, references of the retrieved papers and personal archives of references have been used.

## Results

A total of 30 experimental studies were retrieved, including 25 on humans and 5 on animals. A complete list of these studies, including the type, sample, experimental set-up, controlled variables, and main findings, is reported in Table 1, in chronological order.

**Table 1:** Results of the experimental studies.

**References**	**Year**	**Study**	**Model and sample**	**Experimental setup**	**Controlled variable**	**Findings**
Takahara et al. (21)	1977	RCT crossover study	H; 6 healthy male volunteers	(A) 2.5 g GHB IV; (B) placebo	GH, PRL levels	Increase in plasma GH at 30, 45, 60, and 90 min after injection. The plasma prolactin level increased significantly at 45 and 60 min after GHB injection
Cleghorn et al. (22)	1981	Two RCT double blind	H; 1. 12 patients with chronic insomnia 2. 6 patients with chronic insomnia	(A) 30 mg/kg GHB; (B) placebo	1. Sleep recording, EEG 2. Cortisol, GH, PRL, TSH, LH, EEG	1. Reduction in time awake, REM latency 2. 3-h EEG amelioration, time awake reduction, stage 2 sleep, and slow-wave sleep increase. GH increased in the first 3 h and reduction after. Reduction of cortisol in the first 3 h
Takahara et al. (23)	1981	RCT crossover study	A; male rats	(A) Substance P intraventricular + placebo; (B) Substance P intraventricular + 125 mg/0.5 ml IP GHB	GH, PRL levels	GHB significantly increased serum GH and PRL levels, suppressed by pretreatment with substance P
Gerra et al. (24)	1994	RCT crossover study	H; 9 healthy volunteers	(A) O 1.5 g GHB; (B) O 1.5 g GHBA and IV flumazenil; (C) O placebo	GH and PRL levels	GHBA induced a significant increase in GH plasma levels, prevented by flumazenil pretreatment
Gerra et al. (25)	1995	RCT crossover study	H; 10 healthy volunteers	(A) O 1.5 g GHB; (B) O GHB and IV. naloxone; (C) O GHB and O metergoline; and (D) placebo	Plasma GH levels	GHB induced a significant increase in GH plasma levels, antagonized by metergoline but not by naloxone
Vescovi and Di Gennaro (26)	1997	RCT crossover 3-arms study	H; 20: 10 M cocaine addicts, before and after 30 days of abstinence; 10 controls	(A) O GHB 25 mg/kg, (B) placebo	Serum GH levels	GHB induced a significant increase in plasma GH levels in the normal controls but not in cocaine addicts
Van Cauter et al. (27)	1997	RCT blind study	H; 8 volunteers	Bedtime O placebo, 2.5, 3.0, and 3.5 g of GHB	Polygraphic sleep recordings. GH, PRL, cortisol, TSH, melatonin, IGF-I, and IGFBP-3 blood levels	Increased duration and amplitude of GH release after the sleep onset. Transient elevation in prolactin and cortisol. IGF-I and IGFBP-3 were not altered by GHB administration
Addolorato et al. (28)	1999	Observational prospective case-control (add-on) study	H; 45 alcohol dependent patients, of which 31 reached abstinence with no GHB, 13 with GHB; 25 controls	(A) Psychological support and/or self-help groups, (B) 50 mg/kg/day GHB in addition	Body composition assessed by anthropometry, bioimpedance analysis, and tritiated water method; plasma GH, IGF-1 levels; urinary cortisol, and nitrogen. Assessment at 1, 2, 3, and 6 months of abstinence	GHB did not affect body composition and GH levels compared with controls. Fat-free mass did not differ from baseline
Rigamonti et al. (29)	2000	Multi-treatment and stages RCT study + *in vitro* studies	A; rats and dogs	(1) Acute rat pups: 25, 100, 150, and 300 mg/kg GHB SC vs. baclofen and placebo; (2) acute adult rats 100 mg/kg GHB IP vs. baclofen and placebo, 200 mg/kg GHB vs. NCS-382, GHB + NCS-382 and placebo; (3) conscious rats 1,500 mg/kg GHB IP vs. placebo; (4) conscious rats 10 days 50 mg/kg GHB IP vs. placebo; (5) conscious dogs 20 or 40 mg/kg GHB IV vs. baclofen, clonidine, GH releaser and placebo (6) conscious dogs 50 mg/kg GHB IV vs. hexarelin or both	GH and PRL levels	GHB lacks any GH-releasing activity in rats and dogs
Vescovi and Coiro (30)	2001	RCT crossover, single-blind study	H; 13: 6, 4 years abstinent alcoholic patients; 7 controls	(A) 800 mg sodium valproate, (B) 10 mg baclofen, (C) 25 mg/kg body-weight GHB, (D) placebo	Blood GH levels	GH elevation with all drugs in control. GH elevation in abstinent alcoholics with GHB
Scharf et al. (31)	2003	RCT crossover double-blind study	H; 18 fibromyalgia patients	(A) 6.0 g/day SXB (B) placebo for 1 month	Polysomnography, tender points, pain/fatigue score; subjective sleep scores	Decrease in tender points and pain/fatigue score, reduction in sleep latency and slow-wave (stage 3/4) sleep increase; morning alertness and quality of sleep improvement
Murphy et al. (32)	2007	RCT	(A) Rats with TBSA > 40%	(A) Burn, no drug, (B) burn + 100 mg/kg O GHB, (C) burn + 200 mg/kg O GHB, (D) Burn + 1,000 mg/kg O GHB, (E) Sham, no drug, (F) Sham + 100 mg/kg O GHB	Dual energy x-ray absorptiometry; serum GH and IGF-1; semiquantitative burn wound morphology by microscopy	Incremental elevations in serum GH levels and IGF-1 in burned animals. Burn wounds treated with GHB 1,000 epithelialized significantly more rapidly. No effect on body mass
Nava et al. (33)	2007	RCT	H; 42 alcoholic inpatients	(A) 50 mg/kg four times/day O SXB, (B) 0.5 mg/kg four times/day diazepam for 3 weeks	Withdrawal syndrome, cortisol	Reduced hypercortisolism
Hussain et al. (34)	2009	Observational retrospective study	H; narcolepsy and cataplexy patients treated with sodium oxybate for at least 2 months	4.5–10.5 g SXB/night, mean 6.9 g/night. Mean duration of therapy 25 months (2–76 months)	Pre-sodium oxybate and on-sodium oxybate weight	Decrease in weight of 3.4 kg with SXB, more marked in cataplexy patients; no weight correlation with dose or duration of therapy
Russell et al. (35)	2009	RCT double-blind study	H; 188 fibromyalgia patients	(A) 4.5 g/day SXB, (B) 6.0 g/day SXB, (C) placebo for 8 weeks	Pain rating (visual analog scale), sleep assessment (Jenkins sleep scale), self-reported questionnaires	Improvements in sleep quality and pain
van Nieuwenhuijzen et al. (36)	2010	RCT	A; 48 (rats)	(A) Daily i.p. 500 mg/kg GHB, (B) MDMA, (C) GHB/MDMA, (D) placebo over 10 days	Locomotor activity and body weight and withdrawal effects	All groups (A, B, C) weighed less than placebo (D) at 10 days of treatment and after 7 days
Donjacour et al. (37)	2011	RCT	H; 8 narcolepsy-cataplexy patients, 8 controls	Two times 3 g/night SXB for five consecutive nights	24-h blood sampling for GH levels, IGF-I, IGFBP-3, sleep patterns	SXB increased total 24-h GH secretion rate in narcolepsy patients, but not in controls
Russell et al. (38)	2011	RCT double-blind study	H; 548 fibromyalgia patients	(A) 4.5 g/day SXB (B) 6.0 g/day SXB (C) placebo for 14 weeks	Fatigue and pain rating (visual analog scale), sleep assessment (Jenkins sleep scale), self-reported questionnaires	Improvements in sleep quality, fatigue, and pain
Spaeth et al. (39)	2012	RCT double-blind study	H; 573 fibromyalgia patients	(A) 4.5 g/day SXB, (B) 6.0 g/day SXB, (C) placebo for 2–12 weeks	Pain and fatigue (visual analog scale), sleep assessment (Jenkins sleep scale), self-reported questionnaires, tender points	Improved quality of sleep Improvement, reduction in fatigue, and pain VAS
Vienne et al. (40)	2012	RCT double blind, crossover study	H; 13 healthy volunteers	(A) 30 mg/kg of O SXB, (B) 0.35 mg/kg of baclofen, (C) GABAβ receptor agonist; before an afternoon nap or before the subsequent experimental night	Psychomotor vigilance task, subjective alertness by Karolinska Sleepiness Scale, declarative (unrelated word-pair learning task, and 2-D face-location memory task), and (finger sequence tapping task) procedural memory, polysomnographic and actigraphic recordings	SXB only slightly and transiently negatively affected sustained attention and subjective alertness. A nap under SXB did not differently affect any of the tested memory variables compared with placebo
Rousseau et al. (41)	2014	RCT blind study	H; patients with TBSA > 30%	At the 5th day following injury: (B) evening bolus of 50 mg/kg, (C) continuous infusion at the rate of 10 mg/kg/h (P) or absence of GHB for 21 days	Skin biopsy to assess keratinocytes proliferation rate, IGF1 levels	Higher mean Ki67+ keratinocytes number in group C with respect to B; increase of IGF1 concentrations in group C when compared with group P
Donjacour et al. (42)	2014	RCT	H; 9 narcolepsy-cataplexy patients, 9 controls	2 nighttime doses of 2.25 g SXB and up to maximum 9 g/night for 3 months	Glucose and fat metabolism	SXB had a tendency to decrease peripheral (primarily muscle) insulin sensitivity, while it increased hepatic insulin sensitivity; increases lipolysis
Luca et al. (43)	2015	Multiple RCTs	A (obese control and knock-out for GABA mice)	1: Acute 300 mg/kg i.p. vs. placebo and obese vs. GABA KO mice 2: Four baseline day and four with 300 mg GHB/kg/day orally 3: 300 mg GHB/kg/day For 4 weeks vs. placebo also to GABA KO mice	1: Blood cholesterol, triglyceride, glucose, and free fatty acids; corticosterone, PRL, and GH. 2: Locomotor activity, oxygen consumption (VO_2_) and CO_2_ production (VCO_2_), respiratory exchange ratio (RER), food, and water intake, and heat production. 3: Lean mass, fat mass, and water content, weight and metabolome analysis	1: Corticosterone increase after GHB; 2: Decrease in respiratory exchange ratio (fatty acids are preferred to glucose as energy substrate under GHB) 3: No significant differences between treated and control groups for fat mass, lean mass, or water content after 4 weeks of treatment, but a more balanced distribution of fat mass and lean mass; less gain weight in obese mice treated
Bosch et al. (44)	2015	RCT crossover study	H; 16 healthy volunteers	(A) 20 mg/kg of O SXB, (B) placebo	1. Mood effects by visual-analog-scales and the GHB-Specific-Questionnaire; prosocial behavior; reaction time, memory, empathy, and theory-of-mind. 2: GHB, oxytocin, testosterone, progesterone, cortisol, aldosterone, dehydroepiandrosterone, adrenocorticotropic-hormone plasma levels	Stimulating and sedating effects. Basal cognitive functions not affected. GHB increased plasma progesterone, while oxytocin and testosterone, cortisol, aldosterone, DHEA, and ACTH levels remained unaffected.
Van Schie et al. (45)	2016	Observational longitudinal cohort case-control study	H; 26 narcolepsy patients, 15 healthy controls	4.5–9.0 g/day (mean 5.5 g) SXB per day.	Actigraphy and vigilance test battery	SXB was associated with a lower Sustained Attention to Response Task error count (in patients with narcolepsy), but no changes in Psychomotor Vigilance Test reciprocal reaction times. Improvement of sustained attention and a better resistance to sleep.
Brailsford et al. (46)	2017	Non-controlled trial	H; 12 healthy volunteers (6F, 6M)	Bedtime 25 mg/kg GHB	Serum GHB and GH concentration	Absolute increase in GH in male and females returning to basal concentrations at ~90–120 min post-administration
Filardi et al. (47)	2018	Observational longitudinal cohort study	H; 24 narcolepsy pediatric patients	1 + 1 g taken at bedtime SXB	Anthropometric features, questionnaires at 1 year	Significant reduction in BMI after 1 year
Schinkelshoek et al. (48)	2019	Observational retrospective case-control study	H; 81 narcolepsy patients	(A) SXB, (B) modafinil	Anthropometric up to 6 years	Significant BMI decrease with (A) and not with (B)
Dornbierer et al. (49)	2019	RCT, double-blind, placebo-controlled, cross-over study	H; 15 healthy volunteers	(A) 20 and 35 mg/kg O GHB, (B) placebo	Performance and conflict monitoring in the Eriksen–Flanker task	Behavioral analysis revealed prolonged reaction times and unaffected error rates or post-error slowing under GHB. Disrupted performance monitoring but enhanced conflict detection under GHB
Ponziani et al. (50)	2021	Observational retrospective case-control study	H; 129 narcolepsy pediatric patients	(A) SXB, (B) SXB combined with other drugs, (C) other drugs	Anthropometric measures at 1, 2, 3, and 4 years	At 1 year, (A) and (B) showed a significant BMI z-score reduction compared with baseline, which continued for (B) during the second year and then stopped

*RCT, randomized controlled trial; Model and sample: A, animal; H, human; F, females; M, males; TBSA, total burn surface area; Experimental set-up: IP, intraperitoneal; IV, intravenous; O, oral; SC, subcutaneous; GHBA, gamma-hydroxybutyric acid; SXB, sodium oxybate; Controlled variables: EEG, electroencephalogram; IGF-1, insulin-like growth factor I; IGFBP-3, insulin-like growth factor–binding protein-3; LH, luteinizing hormone; PRL, prolactin; TSH, thyrotropin; GHB, gamma or γ-hydroxybutyrate; GH, growth hormone; REM, rapid eye movement; MDMA, 3,4-methylenedioxymethamphetamine; GABA, gamma-aminobutyric acid*.

Most studies corresponded to Randomized Controlled Trial (RCT), with or without a crossover design and the use of placebo or blind/double blind design. Particularly, four studies on fibromyalgia were included, due to the analysis of pain, fatigue, and sleep improvement. One study was a non-controlled trial, six studies were observational, equally distributed between retrospective and longitudinal. Human studies were performed on healthy volunteers, patients with insomnia, or fibromyalgia, narcoleptic patients under sodium oxybate treatment (SXB), including also studies on a pediatric population, and alcohol/substances dependent patients.

Doses of GHB and SXB changed from one study to another, according to the animal/human model, the route of administration and the experimental design. Overall, in humans, GHB/SGB doses tended to be included between 0.8 and 10.5 g, or between 25 and 50 mg/kg, with lower doses in the pediatric population.

Main controlled variables included several hormonal levels, mostly of GH and PRL, but also of cortisol, IGF-1, and many others. Sleep recording and polysomnography were common, also in combination with GH levels to check for an association between variables, or substituted by actigraphy, a validated method to assess sleep parameters. Recently, the latter was also associated with psychomotor abilities test batteries (45). Assessment of body composition and weight was sometimes identified, and performed by means of anthropometry, bioimpedance analysis, and tritiated water method dual-energy x-ray or by simply weighing the body or calculating the BMI. Two articles also performed an extensive and complex evaluation of glucose and lipid metabolism under SXB/GHB treatment (42, 43).

Epidemiological studies and case reports/series are summarized in Table 2. Overall, 24 articles reported the use of GHB or GHB-containing compounds for reasons connected to the enhancing of performance, with a sample number ranging from 1 to 215. When the sample was larger, single information on users were mostly not extractable. The age of users ranged from 17 to 77, with a predominance of around 30 years old, although this could be a bias connected to the study design, which sometimes targeted specifically students. Self-administered doses are often unspecified, the use of a multiple of a “teaspoon” unit or of sips or bottles being reported, with units for retail sale ranging from 30 to 250 g (54). The use of GHB to ameliorate sleep or to resolve insomnia was reported from 1991 to the present. Bodybuilding, ergogenic, and anabolic effects were commonly listed in the desired effects until 2000–2001. Afterward, euphoria, as well as a sensation of increased energy, were more frequently listed as reasons for use (74). Weight loss was both described as a desired and unwanted effect. Curiously, several papers reported the enhancement of communication or of sexual performances as a desired effect, but this was also listed as an unwanted side effect in terms of risky behavior for sexually transmitted infections. Pleasant mood, relaxation, getting rid of daily worries and self-confidence were also listed, as an incentive for use. Finally, the use of GHB as a club drug emerged in connection with the amelioration of dance performance among the desired effects (74).

**Table 2:** Epidemiological and real-case studies.

**References**	**Year**	**Cases**	**Age (years)**	**Dose of GHB/SXB**	**Desired, pleasant effects or scope of use**	**Undesired, unpleasant or side effects**
Dyer (9)	1991	16 (2F; 14M)	17–47	1/4–6 teaspoons	Health-food to aid-dieting and bodybuilding	Weakness, confusion, incontinence, hallucinations, incoordination, tachycardia, agitation, sedation, seizure
Adornato and Tse (51)	1992	1, M	40	1–2 teaspoons (2.5–5 g) until 115 mh/kg	Health-food	Seizure
Chin et al. (52)	1992	6 (3F; 3M)	Mean 40 (23-77)	1 scoop or 1 teaspoon-4 teaspoons	Bodybuilding, GH release, to improved muscle tone, soporific, party drug	Vomiting, drowsiness, difficulty breathing, uncontrolled twitching or shaking, headache, nausea, diarrhea, confusion, dizziness, “high” feeling, intermittent lack of bladder control, unresponsiveness, coma
Luby et al. (53)	1992	6 of 28 gymnasium sold GHB, 22 GHB users interviewed (73% M)	Men 30 (23-40)	1 teaspoon (2.5 g)	Reasons for use: body building (55%); sleep induction (27%), weight losing (14%), euphoria (5%)	Effects reported: drowsiness (73%), high feeling (46%), dizziness (41%), increased sexual arousal (32%), nausea (27%), unconsciousness (14%), loss of peripheral vision (9%)
Galloway et al. (54)	1997	8 cases (2F; 6M)	Mean 30 (22-40)	1 teaspoon/2.5–50 g per day. Also one bottle (30–250 g)	Anabolic effect, to complement exercise program. Relaxation, increased libido and marked euphoria. Amelioration of the after-effects of stimulants. Talkative effect, sleep-induction “heroin-or alcohol-like”	Withdrawal with doom feeling, tremor, insomnia, and anxiety. Vomiting, dysarthria, and discoordination, blacking-out, apnea, and loss of consciousness. Increasing dose, feeling light-headed and tremors, with inability to perform fine motor work. In combination with MDMA agitation, and psychosis
Gruber and Pope (55)	2000	75F athletes. GHB was used by 5 using steroids (20%) and 1 (2% non-using steroid)	–	–	Ergogenic substance	–
Ingels et al. (56)	2000	3 cases (M)	Mean 39 (28-50)	1–4 capful/teaspoons	Club–drug, alcohol and mood enhancer, bodybuilding	Vomiting, seizure, agitation, blurred vision, amnesia, unresponsiveness
Sia and Wong (57)	2000	1 (M)	39	–	Bodybuilding work–out	Drowsiness and convulsion
Dyer et al. (58)	2001	8 cases (2F; 6M)	Mean 26 (22-38)	Daily dose 43–144 g; 1–5 capful every 0.5–3 h	Bodybuilding, anabolic effects, “to help with focus,” euphoria, alcohol substitution	Withdrawal with intermittent anxiety, tremor, hypertonia, myoclonic jerks, nausea, vomiting, diaphoresis, tachycardia, hypertension, paranoid feelings, visual and auditory hallucinations, agitation requiring restraint, loss of short memory, blurred vision, blackouts, confusion, death
Sivilotti et al. (59)	2001	5 cases (1F, 4M)	Mean 26.6 (23-33)	2 sips twice a day-−0.5 every 3 h GHB/GBL	Bodybuilding, supplement exercise	Withdrawal with tachycardia, hypertension, paranoid delusions, hallucinations, and rapid fluctuations in sensorium. Reasons to discontinue: rebound insomnia, mood changes, gastrointestinal effects, tolerance, tremors
McDaniel and Miotto (60)	2001	5 cases (2F, 3M)	Mean 37 (21-57)	30–40 tablets GBL; 1–2 capfuls every 3 h	Health benefits, alleviate panic attacks, weight training supplement	Withdrawal with anxiety, insomnia, tremor, tachycardia, fatigue, diaphoresis, pruritus, depression, and feeling a seizure, confusion, disorientation, hallucinations, disinhibition, psychomotor agitation, amnesia
Camacho et al. (61)	2004	100 HIV-positive (11F; 89M)	40% between 26 and 39 years	52% of the participants have used a GHB containing dietary supplement at least once in their lives	Increased energy (21%), euphoria (18%), and weight loss (11%).	Insomnia, dizziness, nausea, vomiting, and seizures
Yambo et al. (62)	2004	1, M	26	–	Sleep aid and treatment of panic attacks	Unresponsiveness, convulsions
Zvosec and Smith (63)	2004	48 participants		0.75–120 g/day	19 participants began to use for purported health benefits, including sleep (44%); self-treatment of depression (31%), anxiety (31%), social anxiety (27%), and addiction (13%); weight loss (29%); energy (29%); and bodybuilding (23%).	Withdrawal syndrome
Camacho et al. (64)	2005	215 students (125 F; 90M)	22.8 mean	GHB users reported use 1–2 times per month	With GHB-compounds: euphoria (n = 41), increased energy (n = 51), weight loss (n = 59), and dizziness (n = 13). With GHB: (36/41 88%) euphoria; 2/41 weight loss; increased energy	Irritability (n = 3), a decreased need for sleep (n = 3), and social problems associated with the use of GHB (n = 1)
Perez et al. (65)	2006	1, F	29	GHB every 2–3 h	Sleep aid and intoxicant, past use for aerobic workout	Withdrawal syndrome with tachycardia, agitation, delirium, hypertension, diaphoresis, hallucinations
Bennett et al. (66)	2007	1, M	36	32 capfuls or ounces of GHB per week, every 2 h	Insomnia-alleviating effect and body building properties	Vomiting, difficulties in breathing, loss of consciousness, withdrawal syndrome with shaking, lightheadedness, malaise, agitation, irritation, hyperventilation tremors
Skarberg et al. (67)	2009	15 patients (46.9%) of an addiction center	–	–	Improved sleep, GH release	–
Klötz et al. (68)	2010	5 prisoners (15.2%)	–	–	In addition to steroids as PED	–
Lee and Levounis (69)	2011	17 college campus respondents (1F; 16 M)	Mean 31 (22-50)	Teaspoons, bottle caps, and cubic centiliters (cc's) of liquids 2–3 g	Sexual desire 65%, elevated mood (41%), relaxation (30%), loss of inhibition (30%) anxiolytic, social interaction, improved health (weight loss, toned muscles, and generally better appearance 12%), special senses	CNS depression, seizures, increased appetite, sexual effects
Kapitány-Fövény et al. (70)	2015	60 GHB consumers (20F; 40M)	Mean 25.6	42 participants (70%) have used GHB in the last year	Most beloved effects: pleasant mood (45.3%), increased energy (20.2%), euphoria (18.4%), relaxation (13.5%), and sociability (13.5%). Sexual enhancement and sexual openness. Sexual disinhibition (28.8%), heightened sense of touch (13.8%), and more intense orgasms (12.3%)	Nausea or sickness (45%), blackouts (33.5%), vertigo (20.2%), and fatigue or weakness (13.5%). <10% headache, anxiety, disorientation, hangover, depression, deviant behavior, irritability, bad taste, sweating, and dehydration
Grund et al. (71)	2018	146 GHB consumers on a minimum of 10 episodes (28%F; 72% M)	Mean 28 (15-53)	Poly–drug use pattern. A mean dose of 4.5 ml. The time between dosing varied from 0.5 to 8 h, with a median of 1.5 h	“Feeling more self-confident, being more sociable” (52%) “happiness and euphoria, and having lots of energy” (51%), “the relaxed, happy, and warm high” (46%), “forgetting daily worries, letting go, dampening of emotions” (41%) and “an enhanced sexual response” (38%); “being able to sleep” (after using stimulants), “muscle building”	“Risk of passing out” (48%), bitter taste (47%), the “risk of becoming addicted” (41%), “difficulties in dosing” (26%), “nausea/vomiting” (25%), “short-term memory loss” (25%), GHB's bad reputation (13%) and “dizziness” (13%)
Bosma-Bleeker and Blaauw (72)	2018	180 substance use-disorder patients			Increase in sexual performances. GHB and stimulants users perceive significantly more sexual enhancement than did patients that used alcohol or sedatives; the GHB group perceived less sexual decline than did the other three groups of patients; more perceived risky sexual behavior	
Bulut (73)	2019	1, M	25	3–4 ml/day GHB at most (every 1 h, 1–1.5 ml each time)	Self-confidence and feeling happier, getting rid of anxiety and distress, easier communication, increasing sexual performance	Urticaria with unknown origin, short-term loss of consciousness, feeling sad and depressed, 4kg weight loss and insomnia

*M, males; F, females; GBL, γ-butyrolactone; SXB, sodium oxybate; CNS, central nervous system*.

Adverse effects ranged from urticaria, to headache, weakness, fatigue, irritability, short-term memory losses, nausea, vomiting, loss of weight, diarrhea and incontinence, various grades of central nervous system depression, with dizziness, confusion, hallucinations, incoordination, loss of peripheral vision until unconsciousness, and death. Withdrawal symptoms were commonly described, including a rebound of vomiting, insomnia, anxiety and tremor, psychomotor agitation, or seizures.

## Discussion

After its abandonment as anesthetic agent, GHB was initially introduced in the market for bodybuilding, claiming an anabolic effect, and as a legal nutritional supplement aiding dieting. Our review confirmed that in the past, until the substance was listed among the controlled ones, this application underlined the majority of real case scenarios, resulting in several intoxication cases (9). Although the use in the world of bodybuilding likely decreased over time, the present review highlighted that GHB consumption among users of AAS and of other PEDs is not uncommon also nowadays (5, 55, 57, 59, 63, 68). For example, a publication by Gruber and Pope (55), in the year 2000, highlighted that 20% of woman athletes using steroids also consumed GHB. Bodybuilding was the motivation for use in 63% of cases referred to an emergency department in 2001 for withdrawal syndrome (58). In 2006 and 2007 cases were described with the use of GHB for aerobic workout (65) and bodybuilding properties (66). In 2010 46.9% of the patients of an addiction center and 15.2% of Swedish prisoners responding to surveys/interviews were shown to consume GHB either to improve sleeping or as a PED in addition to steroids (67, 68).

The concept of “performance enhancing” is difficult to define and to distinguish from other kinds of abuse, due to the wide range of aspects to be covered, including image, appearance, athletic, but also self-perception and self-confidence, social or communicative aspects, and sexual performances. The lack of a precise definition, that merges and overlaps with other recreational settings, might partially explain the fact that the issue of the effectiveness of GHB in increasing, and ameliorating performances is still controversial in the literature, and that scientific evidence remains scarce. Considering these limitations, the scope of our study was to provide a toxicological characterization of the use of GHB as a PED, by reviewing the scientific basis as well as desired/undesired effects, patterns of use, and health risks in this peculiar setting.

### Scientific Basis and Molecular Mechanisms

According to experimental studies and real-case scenarios, the use of GHB among bodybuilders or people seeking an increase in the athletic and muscle-related performance is mainly supported by two types of reasons.

The first one is represented by the release of GH, which has been demonstrated for a short time after the injection or oral administration of GHB in several experimental human and animal settings (21–25, 27, 37, 46). GH is a natural peptide hormone involved in body composition and physical performance, and constitutes one of the PEDs banned by WADA, though the ergogenicity of the substance appears to be weak (75, 76). In the case of GHB, the controversy is way more complex, given the fact that even the GH releasing action has been a matter of debate. Indeed, some studies identified the absence of increase in GH, particularly in cases of cocaine addicts and alcoholics (26, 28) as well as in rats and dogs (29), and the mechanisms underlying this GH secretagog action are not yet fully understood. According to the studies, GHB could stimulate the secretion of GH through the effect on the dopaminergic system, by indirect increase of serotonin levels or by direct effect on the hypothalamus where specific binding sites have been found (21). The role of serotonin emerged by the antagonizing effect of metergoline (a serotonin receptor antagonist) pretreatment (25). However, in both GH and prolactin release, GABA should have a role, as demonstrated by the use of GABA antagonists, such as flumazenil, which prevented hormonal release (24). The difference between GHB and other similar GABA-ergic drugs, such as sodium valproate and baclofen, in the ability of inducing a release of GH in 4-year abstinent alcoholics allows one to hypothesize a different neurotransmitter mediation: GH-releasing activity of GHB might be mediated by muscarinic cholinergic neurotransmission, while GABA might act through a dopaminergic route (30).

The conflicting results obtained by Rigamonti and Móller (29), given the flumazenil-mediated effect (24), were interpreted as if the GH-releasing activity of GHB was mainly due to an endogenous conversion into GABA. Moreover, the absence of GH increase in cocaine addicts and alcoholics might be due to a disruption of the GABAergic system (26, 28).

The disruption of the GABAergic neurotransmission might also hamper the emergence of body modification induced by GHB (28). Indeed, no effect was shown of chronic GHB administration on fat and fat-free mass, wait-to-hip ratio or GH levels in alcoholics. However, this could be due to the low doses of GHB, used to treat alcohol dependency, or to an impairment of the hypothalamic–limbic system and GABAergic neurotransmission in the brain of alcoholics (28). It would be interesting to observe whether these changes in body formation might occur after a longer period of abstinence (4 years), which, as demonstrated by Vescovi and Coiro (30), restored the GHB-mediated release of GH.

Interestingly, a GH release was also seen in animals affected by burn wounds for a total body surface over 40%, in which wound morphology analysis testified a more rapid healing process (epithelialization rates and layer thickness) under high doses of GHB treatment compared with controls (32). Higher keratinocytes proliferation rates were also found in humans with severe burn injuries (41). It has to be underlined that, in the context of athletic performances, a potential for the use of GHB might arise from this skin wound healing effect, once confirmed on a larger scale.

The complexity of the GHB-mediated claimed anabolic or ergogenic effects are also highlighted by the controversial effect on IGF-1, which is physiologically produced in response to GH, and is thought to mediate many of its effects. Indeed, plasma IGF-I and IGFBP-3 levels were not altered by GHB administration in Van Cauter, measured 9 h after drug ingestion (i.e., 7–8 h after the major secretory pulse) (27). No difference was also demonstrated in alcoholics (28) and in the study of Donjacour et al. (37). However, IGF-1 was found increased in burnt animals and humans (32, 41).

Results on cortisol were rather conflicting. Some RCTs found a reduction in cortisol in the first 3 h after GHB administration in patients with chronic insomnia, when GH increased (22), while similar studies, investigating sleep or mood effects in healthy volunteers, reported the opposite (27, 44). In alcoholic inpatients chronically administered GHB, hypercortisolism was reduced (33). Interestingly, one case of Cushing syndrome and one of chorioretinopathy, related to GHB intake, have been reported, suggesting an influence of GHB on cortisol and related hormones (77, 78).

The second type of reasons for GHB use, tightly connected to the previous one, is represented by the sleep-amelioration effect, with a shortening in sleep and rapid eye movement (REM) latency, and increase in slow-wave sleep (22). As shown by experimental studies, GHB administration led to an increase in stages III and IV of non-REM sleep compared with placebo, showing an association, both temporal and quantitative, with GH release. Both EEG amelioration and GH level increase lasted approximately 3 h in patients suffering from chronic insomnia (22). The association was confirmed in healthy volunteers, while prolactin and cortisol levels were not associated with the sleep pattern (27), and in hypocretin-deficient narcolepsy patients, treated with SXB twice a night for 5 days (37). Independently from the GH releasing effect, the induction of sleep, a relaxing effect, and the resolution of insomnia problems were frequently listed as reasons for GHB intake within real cases (52, 53, 62, 63, 65–67). For example, the improvement of sleep was associated with a reduction of fatigue and pain in groups of fibromyalgic patients treated for a month at 4.5 and 6 g SXB daily (31, 37, 73, 75, 76). However, fatigue and weakness were also reported among unpleasant effects of GHB intake (70), and this limits the use of GHB as a muscle-related performance enhancer.

Besides this, experimental studies, epidemiological, and real-cases scenarios also accounted for a wide range of reasons to consume GHB in the amelioration of performances, image, and sociability. The drug is consumed most often in combination with other drugs, as demonstrated by a cross-sectional survey in the Netherlands (71), and the performance enhancing effect of GHB does not only include better sleep and muscle growth, but also weight loss, enhanced sexual desire and performance (70, 72), feeling powerful/stronger, better appearance, toned muscles, increased alertness, general improved health diminished acne, improved social bonding, and increased speech (69).

As for weight loss, a first evidence of a scientific basis for this claiming arose in rats treated daily with GHB and even 7 days after discontinuation of the treatment (36). Patients tend to lose weight (34, 47, 50) as demonstrated in the pediatric population after 1 year of SXB in mono-therapy and after 2 years of combined therapy with other drugs, and this might be connected to an enhanced lipolysis and preferential burning of fatty acids (42, 43). The study of Luca et al. (43) also demonstrated a more balanced distribution of fat and lean mass, together with a reduction in gain weight in obese treated mice. Most of this scientific evidence concern obese patients or those with a baseline altered metabolism. Nevertheless, in real cases, it has been shown that weight loss might be also an unintended effect of SXB (73), with a loss up to approximately 30 kg (79).

### Psychoactive and Other Performances

Some subjects in the real cases claimed to use GHB “to help focus” in a specific task (58). In contrast with the positive and desired effects reported by some users concerning attention and alertness (69), a study on healthy volunteers monitoring the performance in psychomotor vigilance task and subjective alertness (Karolinska Sleepiness Scale) showed a reduction in attention and alertness after a nap under SXB, although declarative and procedural memory were not affected (40). However, this effect was only transient and the prolonged nap, with increased sleep inertia, might have had a role in the lower psychomotor performance and subjective alertness test. In narcolepsy patients, reaction times were not affected by SXB, while an improvement of sustained attention, with lower error count in a response task, was observed (45). However, once again the ameliorating effect was noted in patients, which were characterized by worse performance compared with controls. A recent randomized control trial on healthy volunteers, conversely, demonstrated prolonged reaction times with altered performance monitoring (49). This might mean that GHB could impair the inner ability of detecting the commission of errors though increasing the visual salience of external stimuli (49).

Beside this, the psychoactive effect of euphoria, connected to an increased confidence and to the feeling of being powerful/stronger represent a sought effect in GHB users (64, 69, 71–73). For example, in one case described by Galloway et al. (54), GHB was initially consumed for anabolic effects, but this was soon superseded by a desire for euphoria. Indeed, as recently shown among participants to a survey, APEDs not only allowed the enhancement of the body and muscles, but were consumed to obtain a psychological effect, such as self-control, the development of knowledge and expertise, sense of meaning, wellbeing, and improved quality of life (80). Euphoria was listed only by 5% of gym GHB users in 1992, but a marked euphoric effect started to be reported in the following studies, reaching 33.5% of respondents (70), 52% of GHB consumers (71) until 88% of respondents among students in 2005 (64). An enhancing effect was also reported in mood, in sociability and in sense of touch, connected to sexual performances (70). Taken together, these data suggests that the reduction in GHB use among bodybuilders might be more related to an unavailability of the drug in health-food stores than to its inefficacy in enhancing some sort of performance (69).

As reported in previous publications (20), in the real-context scenarios, GHB is associated with negative psychomotor performances related to central nervous system depression with altered or impaired vision, drowsiness, incoordination and inability to stand, impaired alertness, blackouts, and memory losses, loss of consciousness until “falling asleep” in unwanted places (9, 51, 53, 56, 61, 62, 69, 71). All these negative effects suggest that GHB has not an objective ameliorative effect on the main psychomotor and psychoactive performances (alertness, memory, vigilance, etc.), except for subjective feelings of increased energy and wellbeing, sociability, and sexual performances. Nevertheless, these effects in self-confidence, sexual performance, communication, and perceived energy cannot be overlooked when talking about GHB and performances, given the fact that these effects are the most desired among GHB users [52% of GHB consumers in a survey of 2018 (71)]. Moreover, as shown by the study of Grund et al. (71), self-confidence-seeking behavior was associated with more coma experiences and the frequency of GHB use correlated with the “enhancing sexual experience” reason for consumption (70). The amelioration of sexual performances is also a reason for use despite the negative effects associated with GHB, including the potential for loss of consciousness (69).

### Health Risks

The adverse effects of GHB were at first considered “mild,” as demonstrated in the therapeutical setting. Also, some publications of real-cases intoxication stated that, when discontinuing GHB administration, a full recovery is usual, in the absence of long-term sequelae (52). However, symptoms experienced by users consisted also in severe central nervous system depression with apnea, loss of consciousness, coma (54, 56), and death (58). Our review allowed us to point out some considerations underlining a high risk in a recreational setting, and especially in the context of PEDs.

First of all, when GHB is consumed as a dietary supplement, there is no possibility to accurately control the dosage. In the recreational setting, the use of “teaspoons” of product, as indicated by the bottle of nutritional supplement (53), as well as of scoops, sips, bottle caps, or cubic centiliters (cc's) of liquids were reported, and there is no doubt that a dose can hardly be calculated from this assumption (9, 51, 52, 56, 58–60, 69). The difficulties in self-dosing were also reported by users, who stated that they mostly use plastic vials or bottles, while only a minority tried to reach more accuracy with a syringe (71). On the contrary, in the therapeutic setting, monitoring of the dosages and plasma levels is done, in order to limit potential over- or underdosages.

Real cases also suggest that casual users could self-administer much higher doses than what is actually indicated, from half a teaspoon to four or six, followed by the onset of symptoms such as uncontrolled twitching or difficulty in breathing or unresponsiveness (9, 52). In one case, an entire bottle of GHB was consumed daily (54). In one case presenting to the emergency department for withdrawal symptoms, the higher daily dose was estimated at 43–144 g (58).

Withdrawal symptoms could be severe, spanning from anxiety, hypertonia, myoclonic jerks, and tremors, accompanied by insomnia, gastrointestinal symptoms such as nausea and vomiting, cardiovascular complications such as hypertension and tachycardia, and even confusion, amnesia, psychotic agitation, and delirium (54, 58–60, 65, 66).

Changes in the name of products containing GHB and GBL, homemade, or internet-based preparations further reduce the awareness toward the risks arising from GHB consumption (58, 60, 81). In a recent survey, subjects declared among the benefits of GHB the ease of home-producing the drug (71). It is also perceived as a natural and safe alternative (60), given the fact that it is an endogenous substance and due to the release of GH (24% of respondents in a survey of 2011) (69). In one case described by Galloway et al. (54), a 20-year-old user consumed GHB because he was told that it was an amino acid.

Most of the GHB users, although searching for a health food or natural supplier, experienced a “high” sensation, and 46% of 22 users interviewed in six gyms in 1992, once again disclosing the potential for abuse (52, 53). This is further confirmed by the tendency to escalate self-administered doses (52, 53). Due to the short half-life and rapid metabolism and excretion, the prevention of withdrawal symptoms and the tolerance require the escalation of administrations to every 2 to 4 h in a pattern of “around-the-clock” dosing (54, 58–60, 73, 81). The median gap between doses, in a recent survey, was around 1.5 h (71). This is important since the more frequent the GHB administrations, the higher is the risk for coma (71).

A significant risk hazard is also derived from the combination with other licit or illicit drugs (54, 69–71). While some gymnasium users reported no mixing with other drugs (53, 54, 59), other consumers used GHB in combination with other drugs, from alcohol to methamphetamine, heroin, psychedelics, barbiturates, benzodiazepines, and anti-psychotics (54, 58, 69, 71). The polydrug pattern is sometimes connected to the amelioration of symptoms and after effects either induced by GHB withdrawal, e.g., in the case of alcohol, benzodiazepines, or phenobarbital co-consumption (60); also, GHB could be consumed in the attempt to mitigate the adverse effects produced by other-drugs, especially in the case of methamphetamines (54). The combination of LSD and GHB might be sustained by the anxiolytic effect of GHB, leading to a pleasant experience (54).

Although the consumers are mostly young or middle-aged, and the risk for coma is higher when GHB consumption started at a younger age (71), intoxication cases also highlight that the risk is not only confined to young people but also to older ones, sometimes willing to restore their body appearance and muscular tone (52).

## Conclusions

In conclusion, several aspects of the use of GHB as a performance enhancer have been retrieved in the literature, ranging from the more traditional GH release and sleep induction to more recent reasons for use, including the psychoactive subjective effects on mood and energy, and the enhancing of sexual performances. In this view, the term “performance enhancer” should be redefined taking into consideration aspects that transcend only athletic performance. Given the polydrug abuse pattern, the difficulties in the dosages, the tendency to increase the dose until an “around-the-clock” administration is reached, these kinds of recreative consumption appears to be associated with significant health risks.

## Author Contributions

AG, FB, and RG contributed to the conception and design of the study. AG organized the database and wrote the first draft of the manuscript. RG and FB wrote sections of the manuscript. All authors contributed to the manuscript revision, read, and approved the submitted version.

## Conflict of Interest

The authors declare that the research was conducted in the absence of any commercial or financial relationships that could be construed as a potential conflict of interest.

## Publisher's Note

All claims expressed in this article are solely those of the authors and do not necessarily represent those of their affiliated organizations, or those of the publisher, the editors and the reviewers. Any product that may be evaluated in this article, or claim that may be made by its manufacturer, is not guaranteed or endorsed by the publisher.
